# Patterns of Manufacturer Coupon Use for Prescription Drugs in the US, 2017-2019

**DOI:** 10.1001/jamanetworkopen.2023.13578

**Published:** 2023-05-16

**Authors:** So-Yeon Kang, Angela Liu, Gerard Anderson, G. Caleb Alexander

**Affiliations:** 1 Department of Health Policy and Management, Johns Hopkins Bloomberg School of Public Health, Baltimore, Maryland; 2 Center for Drug Safety and Effectiveness, Johns Hopkins Bloomberg School of Public Health, Baltimore, Maryland; 3 Department of Epidemiology, Johns Hopkins Bloomberg School of Public Health, Baltimore, Maryland; 4 Division of General Internal Medicine, Johns Hopkins Medicine, Baltimore, Maryland

## Abstract

**Importance:**

Although manufacturer-sponsored coupons are commonly used, little is known about how patients use them within a treatment episode.

**Objectives:**

To examine when and how frequently patients use manufacturer coupons during a treatment episode for a chronic condition, and to characterize factors associated with more frequent use.

**Design, Setting, and Participants:**

This is a retrospective cohort study of a 5% nationally representative sample of anonymized longitudinal retail pharmacy claims data from October 1, 2017, to September 30, 2019, obtained from IQVIA’s Formulary Impact Analyzer. The data were analyzed from September to December 2022. Patients with new treatment episodes using at least 1 manufacturer coupon over a 12-month period were identified. This study focused on patients with 3 or more fills for a given drug and characterized the association of the outcomes of interest with patient, drug, and drug class characteristics.

**Main Outcomes and Measures:**

The primary outcomes were (1) the frequency of coupon use, measured as the proportion of prescription fills accompanied by manufacturer coupon within the treatment episode, and (2) the timing of first coupon use relative to the first prescription fill within the treatment episode.

**Results:**

A total of 36 951 treatment episodes accounted for 238 474 drug claims and 35 352 unique patients (mean [SD] age, 48.1 [18.2] years; 17 676 women [50.0%]). Among these episodes, nearly all instances (35 103 episodes [95.0%]) of first coupon use occurred within the first 4 prescription fills. Approximately two-thirds of treatment episodes (24 351 episodes [65.9%]) used a coupon for the incident fill. Coupons were used for a median (IQR) of 3 (2-6) fills. The median (IQR) proportion of fills with a coupon was 70.0% (33.3%-100.0%), and many patients discontinued the drug after the last coupon. After adjustment for covariates, there was no significant association between an individual’s out-of-pocket costs or neighborhood-level income and the frequency of coupon use. The estimated proportion of fills with a coupon was greater for products in competitive (19.5% increase; 95% CI, 2.1%-36.9%) or oligopolistic (14.5% increase; 95% CI, 3.5%-25.6%) markets than monopoly markets when there is only 1 drug in the therapeutic class.

**Conclusions and Relevance:**

In this retrospective cohort analysis of individuals receiving pharmaceutical treatment for chronic diseases, the frequency of manufacturer-sponsored drug coupon use was associated with the degree of market competition, rather than patients’ out-of-pocket costs.

## Key Points

**Question:**

When and how frequently do patients use manufacturer-sponsored coupons for their prescription drugs during a treatment episode?

**Findings:**

In this cohort analysis of 35 352 individuals receiving pharmaceutical treatment for chronic diseases, nearly all of the first coupon use occurred with the first prescription fills. The frequency of manufacturer drug coupon use was associated with drugs operating in competitive environments but not with patient’s out-of-pocket costs or the characteristics of neighborhoods where the patients reside.

**Meaning:**

These findings suggest that manufacturer-sponsored coupons were more likely used to initiate the treatment, and the frequency of coupon use was associated with market competition but not patients’ out-of-pocket costs.

## Teaser

This cohort study examines when and how frequently patients use manufacturer coupons during a treatment episode for a chronic condition and examines the factors associated with more frequent use.

## Introduction

Prescription drug coupons are commonly used by patients to offset their out-of-pocket prescription drug costs.^1^ A recent analysis of pharmacy claims found that coupon use results in a nearly 85% reduction in patients’ out-of-pocket costs.^1^ Other studies have examined the factors associated with the availability of manufacturer-sponsored coupons,^2^ the association of coupon use with adherence,^3,4,5^ and spending on drugs.^6^ Also, some studies^7,8^ found that coupon use can induce demand for brand-name drugs by 60% or more by reducing the sales of generic drugs.

Manufacturer-sponsored coupons are available for a variety of conditions, ranging from anti-infectives to chronic diabetes treatment.^9^ These coupons are often distributed to patients through manufacturers’ website or portals for copayment saving programs and are used at the point of sale. Despite the prevalent use of coupons, we could not identify any studies that characterize how individuals actually use coupons within an episode of care. This is important because pharmaceutical manufacturers can offer coupons with various terms of use and conditions, depending on their commercial interests. For example, some manufacturer coupons are valid for only a single use or specific period following the initial fill, whereas others may automatically renew or stipulate that a discount can be applied to a certain number of fills, regardless of when they occur. Understanding how coupons are used within treatment episode may help physicians and patients understand the availability of coupons when selecting treatment options, as well as to inform prescription drug policies.

We used a nationally representative 5% sample of anonymized, patient-level pharmacy claims to examine patterns of coupon use for prescription drugs treating chronic conditions among commercially insured patients. We focused on when and how often patients use coupons and patient, product, and drug class characteristics associated with such use.

## Methods

This cohort study was exempt from the need for informed consent and an institutional review board at the Johns Hopkins Bloomberg School of Public Health because it did not constitute human participants research, in accordance with 45 CFR §46. This study followed the Strengthening the Reporting of Observational Studies in Epidemiology (STROBE) reporting guideline for observational studies.^10^

### Data

We performed a retrospective cohort study using IQVIA’s Formulary Impact Analyzer between October 2017 and September 2019.^11^ The Formulary Impact Analyzer database is a nationally representative patient-level, transactional claims database sourced from 95% of the retail pharmacies in the US. We focused on prescription drugs with at least 1 transaction where a product-specific manufacturer-sponsored copayment program was used as the secondary method of payment. This identification strategy was validated through consultation with the data provider, IQVIA, and was used in the previous peer-reviewed research.^1^

We supplemented the claims data with 3 data sources. First, we used IBM Micromedex Red Book^12^ to derive drug characteristics, including the availability of generic alternatives, and whether the drug’s primary indication was for an acute or chronic condition. The Red Book database defines drugs treating chronic conditions as drugs with low probability for dosage or therapy changes, commonly used to treat chronic conditions, and usually administered continuously. Second, we used IQVIA’s Uniform System of Classification to define the market of direct competitors according to the mechanism of action for each product of interest (eg, dipeptidyl peptidase 4 inhibitors). The Uniform System of Classification is therapeutic classification of pharmaceutical products considered to compete in the same market and is widely used as the standard in North America.^2,13,14^ Finally, we supplemented the data with information on county income and racial characteristics obtained from the 2019 Health Resources & Services Administration Area Health Resource Files. Detailed methods regarding the data are described in the eAppendix in Supplement 1.

### Study Sample

Manufacturer-sponsored coupons are concentrated in a small number of drugs, as reported in the prior research.^1^ Consequently, many drugs did not have enough treatment episodes to observe significant variation in coupon use patterns among patients within the drug. To address the issue, we first identified drugs that had at least 100 coupon fills reported in the data. Then, we focused on drugs treating chronic conditions because drugs treating acute illnesses were typically administered only once and, therefore, had insufficient prescription refills to observe patterns of coupon usage over the treatment episode; 113 unique drugs treating chronic conditions met the inclusion criteria.

Our unit of analysis was a treatment episode, which we defined as an episode of care where a product used to treat a chronic condition was initiated between January 2018 and October 2018 and that specific drug was not used during the prior 90 days. The treatment episode ended if no more refills were reported with the drug for the patient or our observation period (12 months) ended. Shorter use of drugs, such as those with only 1 or 2 fills with a coupon, accounted for 59.1% of the initial cohort of 90 431 episodes. These treatment episodes may represent a drug trial or immediate drug discontinuation or drug switch. Also, including the one-and-done or two-and-done episodes in the sample would inflate the outcome variables measuring frequency of coupon use within the treatment episode as the percentage of fills with a coupon. These analyses were focused on continuous treatment episodes, which we define as treatment episodes filling 3 or more fills. Detailed rationale and methods for sample selection strategy are described in the eAppendix and eFigure in Supplement 1.

### Explanatory Variables

We quantified the frequency of coupon use as a function of patient, product, and drug class characteristics. For patient characteristics, we included sex, unique products filled, the dollar level of out-of-pocket cost per claim before coupon application, and patients’ neighborhood characteristics (ie, the median household income and percentage of Black residents) in the county where the prescription drug was dispensed.

For product characteristics, we included whether it was a single-source brand-name product, an orphan drug status, and the drug’s out-of-pocket cost relative to the mean of drugs in the same drug class. For drug class characteristics, we included the level of competition within the drug class measured as the Herfindahl-Hershman Index,^15^ an established measure of market concentration, and the mean patient cost-sharing before offset to examine whether market characteristics influenced use of coupons. These market variables were constructed according to the full data set before the sample selection as reported in the prior study.^2^ A detailed rationale and methods for constructing explanatory variables are provided in the eAppendix in Supplement 1.

### Outcome Variables

We examined 2 outcomes. The first outcome of interest was the frequency of coupon use during a given treatment episode, which we computed as the proportion of prescription fills purchased with a coupon of the total number of fills within the treatment episode. In descriptive analyses, we dichotomized treatment episodes into 2 groups: infrequent coupon use (coupons used for fewer than two-thirds of treatment episode fills) and frequent coupon use (more than two-thirds of fills). In the multivariable regression examining characteristics associated with coupon use, this outcome was treated as a continuous measure.

The second outcome of interest was the timing of the first coupon use within the treatment episode to examine whether coupons are used to fulfill the prescription for the new treatment, which is known as a measure of primary drug adherence.^16^ In examining this outcome, we dichotomized individuals into those using a coupon during the first fill of their treatment episode and those who did not.

### Statistical Analysis

We analyzed the data from September to December 2022. First, we examined the unadjusted association between patient (age, sex, prescription number, out-of-pocket cost, and neighborhood income and race), product (mean cost-sharing, total sales for that drug, and orphan drug status), and drug class (mean out-of-pocket cost within class and total sales within drug class) characteristics.

Second, a linear, multivariable, multilevel regression with product and drug class as 2 levels of the data was used to examine the adjusted associations of interest on coupon use, which is defined as a continuous measure. We included all of the variables mentioned earlier and assessed goodness of fit and significance of adding interaction terms using the Akaike Information Criterion and log-likelihood value. We calculated the variance inflation factor for each explanatory variable to evaluate multicollinearity. We clustered SEs at the drug-class level to account for potential correlation among drugs within the same drug class. In sensitivity checks, we tested income and Black resident deciles as additional clusters in the regression.

To estimate the magnitude of the association between each variable of interest and frequency of coupon use, the mean percentage point change in the frequency of coupon use associated with a unit change in a given explanatory variable was assessed. Results were considered statistically significant at 2-tailed, unpaired *P* < .05. All analyses were performed using Stata statistical software version 16 (StataCorp, LLC).

## Results

Of a total of 90 431 treatment episodes, the sample contained 36 951 continuous treatment episodes (40.9%), for a total of 238 474 transactions and 35 352 patients (mean [SD] age, 48.1 [18.2] years; 17 676 women [50.0%]). The median (IQR) number of fills per treatment episode was 5 (4-9) fills, and coupons were used in a median (IQR) of 3 (2-6) fills. Approximately one-third of the treatment episodes (12 120 treatment episodes [32.8%]) included in the sample used coupons for every fill.

Table 1 depicts products commonly associated with manufacturer-sponsored coupon use. The most common uses were for acne (eg, adapalene-benzoyl peroxide), HIV (emtricitabine-tenofovir), asthma (budesonide-formoterol), diabetes (empagliflozin, insulin glargine, and sitagliptin), and thromboembolic disease (rivaroxaban).

**Table 1.** Drug Products Commonly Associated With Manufacturer-Sponsored Coupon Use, 2018-2019

Drug class	In-class frequency of coupon use, mean (SD), %	Product example, brand name (generic name)
Acne treatments without anti-infectives	82.9 (23.9)	Epiduo Forte (adapalene-benzoyl peroxide)
HIV antiviral combinations	72.7 (29.5)	Truvada (emtricitabine-tenofovir disoproxil)
Ophthalmic immunologic	70.4 (30.4)	Xidra (lifitegrast)
Inhaled bronchial corticosteroids	67.1 (29.3)	Symbicort (budesonide-formoterol)
Sodium-glucose cotransporter 2 inhibitors	69.6 (29.3)	Jardiance (empagliflozin)
Analogs of human insulin	58.0 (29.4)	Lantus Solostar (insulin glargine)
Anticoagulants, other	47.4 (33.5)	Xarelto (rivaroxaban)
Dipeptidyl peptidase 4 inhibitors	46.6 (29.7)	Januvia (sitagliptin-phosphate)

Coupons were most commonly used during the initial fill, with approximately two-thirds of treatment episodes (24 351 episodes [65.9%]) using a coupon for the incident fill, and 35 103 first coupons (95.0%) being used within the first 4 fills of a given episode. The median (IQR) proportion of fills with a coupon was 70.0% (33.3%-100.0%).

### Unadjusted Association of Coupon Use With Treatment Episode Characteristics

Table 2 shows the unadjusted association between characteristics of treatment episodes and frequency of coupon use. There were no notable differences in the frequency of coupon use according to patient age and patient sex. Coupon use was less frequent among individuals taking multiple drugs (3619 infrequent coupon users [22.4%] vs 3296 frequent coupon users [31.1%]). The patient out-of-pocket cost before coupon use was slightly higher among those with frequent coupon use (mean [SD], $152.20 [$380.30]; median [IQR], $60.00 [$25.00-$125.70]) than those with infrequent use (mean [SD], $140.60 [$393.40]; median [IQR], $40.00 [$1.20-$111.00]).

**Table 2.** Characteristics of Treatment Episodes by Frequency of Coupon Use, 2018-2019^a^

Characteristics	Patients or products, No. (%)		
	Any coupon use (N = 36 951)	Frequent coupon use (n = 21 784)	Infrequent coupon use (n = 15 167)
Patient characteristics			
Age, mean (SD), y	48.1 (18.2)	46.2 (18.0)	50.9 (18.2)
Sex			
Female	19 826 (53.7)	11 967 (54.9)	7860 (51.8)
Male	17 123 (46.4)	9817 (45.1)	7307 (48.2)
Patients using multiple drugs	8851 (25.4)	3619 (22.4)	3296 (31.1)
Product characteristics			
Out-of-pocket cost per claim before coupon, median (IQR), $	50.0 (15.0-125.0)	60.0 (25.0-125.7)	40.0 (1.2-111.0)
Single-source branded products	29 052 (78.6)	17 041 (78.2)	12 011 (79.2)
Orphan drug designation	1424 (4.7)	851 (5.0)	572 (4.4)
Drug class characteristics			
Out-of-pocket cost within class, median (IQR), $	108.5 (76.3-145.9)	115.5 (76.3-145.9)	94.6 (76.3-145.9)
In-class competition			
Monopolistic competition	14 202 (38.2)	7046 (32.3)	7056 (46.5)
Oligopolistic competition	20 035 (54.2)	12 937 (59.4)	7098 (46.8)
Competitive market	2814 (7.6)	1801 (8.3)	1013 (6.7)

^a^ Data were obtained from the IQVIA Formulary Impact Analyzer, 2018-2019. For descriptive purposes, the frequency outcome of proportion of coupon use in a treatment episode was dichotomized into 2 groups: (1) frequent coupon use, defined as 66% or more of fills, and (2) infrequent coupon use, defined as less than 66% of fills. This cutoff was determined by the mean proportion of coupon use in a treatment episode.

Treatment episodes reported in neighborhoods with the highest decile in the share of Black residents had 7.0% higher coupon use frequency compared with those in the lowest decile (mean [SD], 68.1% [32.5%] vs 61.1% [31.0%]). However, the incremental coupon use frequency per race and income decile was small, and there was not a dose response (eTable 1 in Supplement 1).

Treatment episodes with frequent coupon use were more prevalent among products in competitive markets than those in monopolistic markets (19.5% increase; 95% CI, 2.1%-36.9%). Similar findings were observed on unadjusted analysis when examining the association of patient, product, and drug class characteristics with the timing of coupon use (Table 3).

**Table 3.** Characteristics of Treatment Episodes by Timing of Coupon Use, 2018-2019^a^

Characteristics	Patients or products, No. (%)		
	All coupon use (N = 36 951)	Incident fill with coupon (n = 24 344)	Incident fill without coupon (n = 12 607)
Patient characteristics			
Age, mean (SD), y	48.1 (18.2)	47.6 (18.3)	49.1 (18.1)
Sex			
Female	19 826 (53.7)	13 097 (53.8)	6731 (53.4)
Male	17 123 (46.4)	11 247 (46.2)	5876 (46.6)
Patients using multiple drugs	8851 (25.4)	5773 (24.6)	3546 (28.7)
Patient out-of-pocket cost per claim before coupon, median (IQR), $	50.0 (15.0-125.0)	50.0 (10.0-120.0)	50.0 (25.0-133.3)
Product characteristics			
Single-source branded products	29 052 (78.6)	19 366 (80.0)	9686 (76.8)
Orphan drug designation	1424 (4.7)	922 (4.8)	502 (4.7)
Drug class characteristics, out-of-pocket cost within class, median (IQR), $	108.5.6 (76.3-145.9)	115.5 (77.4-145.9)	82.0 (76.3-143.8)
Drug class competition			
Monopolistic competition	14 202 (38.2)	9191 (37.8)	4911 (39.0)
Oligopolistic competition	20 035 (54.2)	13 257 (54.5)	6778 (53.8)
Competitive market	2814 (7.6)	1896 (7.8)	918 (7.3)

^a^ Data were obtained from the IQVIA Formulary Impact Analyzer, 2018-2019.

### Adjusted Association of Coupon Frequency With Treatment Episode Characteristics

Table 4 depicts the adjusted association of patient, product, and drug class characteristics with frequency of coupon use. The values represent the adjusted difference in the estimated proportion of fills with a coupon during a treatment episode among different subpopulations. For example, the estimated proportion of fills with a coupon was 4.1% (95% CI, 2.7%-5.5%) greater among individuals using 2 or fewer drugs than individuals taking 3 or more drugs, holding other variables constant. Similarly, the estimated proportion of fills with a coupon was greater for treatment episodes for orphan than nonorphaned products (3.1%; 95% CI, 0.6%-5.5%). The estimated proportion of fills with a coupon was greater for products in competitive (19.5%; 95% CI, 2.1%-36.9%) or oligopolistic (14.5%; 95% CI, 3.5%-25.6%) markets than markets when there is only 1 drug in the therapeutic class. After adjusting for other covariates, neighborhood-level income and share of Black residents were not factors significantly associated with coupon use frequency, and these results were consistent in the sensitivity checks by drug class (eTable 2 in Supplement 1).

**Table 4.** Adjusted Association of Coupon Use Frequency With Patient, Drug, and Drug-Class Characteristics, 2018-2019^a^

Characteristics	Adjusted difference in frequency of coupon use, % (95% CI)	*P* value
Patient characteristics		
Patients using ≤2 drugs	4.2 (2.8 to 5.5)	<.001
Patient out-of-pocket cost per claim before coupon (per 10% increase)	0.1 (−0.1 to 0.1)	.42
Neighborhood-level income	−0.1 (−0.2 to 0.1)	.30
Share of Black residents	−0.1 (−0.3 to 0.0)	.05
Product characteristics		
Single-source brand-name drugs	0.5 (−2.1 to 3.1)	.70
Orphan designation status	3.1 (0.6 to 5.5)	.01
Drug class characteristics		
In-class mean out-of-pocket cost per claim (per 10% increase)	0.7 (−0.1 to 1.6)	.10
Oligopolistic market (vs near monopoly)	14.5 (3.5 to 25.6)	.01
Competitive market (vs near monopoly)	19.5 (2.1 to 36.9)	.03

^a^ Data are from the IQVIA Formulary Impact Analyzer, 2018-2019. Estimates are derived from a multilevel regression model of the coupon use frequency among commercially insured patients to account for clustering by drug class. Covariates included patient out-of-pocket cost per claim, in-class out-of-pocket cost per claim, sex, neighborhood characteristics (ie, the median household income and percentage of Black residents) in the county where the prescription drug was dispensed, the prevalence of coupon use among the in-class competitors, the number of prescription fills of the treatment episode, revenue size of the manufacturer, and market size. The median (IQR) proportion of fills with a coupon per treatment episode was 70.0% (33.3%-100.0%).

### Adjusted Association of Timing of Coupon Use With Treatment Episode Characteristics

eTable 3 in Supplement 1 depicts the adjusted association of each variable with the timing of the first coupon use. For example, the odds of coupon use for the first fill of a treatment episode were significantly greater for single-source, branded products than their counterparts (odds ratio [OR], 1.7; 95% CI, 1.4-2.1). Similarly, the odds of coupon use during the first fill of an episode also were greater for orphan than nonorphan products (OR 1.2; 95% CI, 1.0-1.5), products with a higher mean out-of-pocket cost per claim (OR, 1.9; 95% CI, 1.2-2.9), and products in competitive markets (OR, 3.4; 95% CI, 1.4-8.1) but not those in oligopolistic (OR, 1.6; 95% CI, 0.9-2.8) markets, compared with monopolistic markets.

## Discussion

Although drug coupons are frequently advertised to consumers,^17,18^ their continuous availability is often vaguely reported in small print.^19,20^ Some manufacturers offer coupons for drugs that can be used indefinitely,^21^ whereas other drugs have no mention of the renewability of the coupon.^20,22,23^ To our knowledge, the findings of this cohort study on coupon use frequency and timing provide the first evidence on how manufacturer-sponsored coupons are actually used among patients using prescription drugs for their chronic conditions.

This study builds on existing literature showing that coupons are provided to serve manufacturers’ commercial interests. Manufacturers’ decisions to provide coupons can have distinct but interrelated impacts on patient, payers, and health systems. For price-sensitive patients, coupons can lower their out-of-pocket expenses in the short run and enable them to fill their prescriptions. However, for payers, these coupons can increase demand for high-cost drugs and place a greater financial burden on prescription drug spending. Furthermore, manufacturers may be disinclined to offer universal rebates for the drug to payers if they can generate revenue by targeting individual patients with coupons. If it is the case, manufacturers may have less incentive to reduce prices, resulting in a greater financial burden on health systems. Our findings shed light on the coupon use patterns in this intersection between pharmaceutical business and policy.

In-class mean frequency of coupon use differed across drug classes and individual products. Prior research^2^ suggested that manufacturers’ coupon offering is associated with the prevalence of coupons and entry of similar products in the drug class, not with the level of patient copayment. In addition, after adjusting for covariates, patients’ coupon use frequency was significantly associated with the level of market competition facing a given drug rather than patient characteristics.

Given that coupons yield a high return on investment to manufacturers,^24^ this finding suggests that manufacturers selectively provide coupons in a continuous fashion depending on the competitive landscape and profit potentials. In other words, although more continuous use of a coupon insulates individuals from out-of-pocket costs and could increase adherence to that treatment regimen, drugs without alternatives in the same class are likely to have fewer coupons. Physicians should be aware of which drugs are most likely to continually offer coupons through the episode of care and should consider electing to use the lower cost drug without coupons to avoid potential disruptions in care that might occur if coupons are not subsequently available to patients.

Our finding that coupons are more likely to be used on a first fill than a subsequent fill during a treatment episode aligns with the notion that manufacturers use coupons to introduce patients to new products. Because filling the first prescription is an essential indicator of primary drug adherence, the coupon use for the first fill may offer advantages for patients who may be hesitant to initiate the treatment and for manufacturers seeking to attract new consumers.

Our findings are relevant to policy debates around prescription drug coupons.^17^ Several policies have been proposed or introduced by individual states, either to improve the benefit of coupons or to reduce inefficiencies in the pharmaceutical marketplace. Massachusetts and California prohibit manufacturers from distributing coupons for brand-name drugs when there are generic alternatives.^25,26^ However, these interventions leave drugs without generic competitors outside the regulations. Although policy makers are unlikely to ban coupon use for privately insured individuals, as is done for publicly insured patients,^27^ policies that improve access to coupons among patients in need, while simultaneously reducing their greatest market-distorting effects, should be considered.

### Limitations

Our study has several limitations. Our data cannot explain why patients discontinued coupon use, which might be the result of a combination of patient, physician, and manufacturer factors. Also, the data did not include patients’ insurance plan design (eg, high-deductible plans) and coverage for specific drugs, which may affect the patients’ drug access and affordability and demand for coupons.

Because the terms and conditions of each coupon are often vaguely defined and subject to change, we were unable to identify a reliable source of data to determine their exact association with coupon usage. To overcome this limitation, we took the actual use of the coupons as a proxy measure of manufacturers’ decisions, which are often driven by the market. Future research exploring the variation in the terms and their effects on coupon use is needed.

Our study’s results are applicable to manufacturer-sponsored coupons only and cannot be generalized to other forms of copayment assistance programs, such as independent charity patient assistance programs or disease-specific funds.^28^ Although these programs may have distinct terms owing to their charitable purposes, actual use data were unavailable. Future research should investigate how the use of these programs differs.

Our analysis defined direct competitors according to mechanisms of action based on prior research.^2^ However, this approach may have some limitations in capturing coupon use associated with interclass switching and broad competition among drugs used for the same indication.

This study did not include drugs used to treat acute conditions or one-and-done treatment episodes because of the short-term nature of drug use, which may have different factors associated with the coupon usage. The study did not examine outcomes of the coupon use patterns, such as the association of coupons with switching or discontinuation of drugs. Further research in this area is warranted. In addition, this study’s focus was limited to the pre–COVID-19 pandemic period; thus, it does not provide insights into how coupon usage patterns may have been affected by the public health crisis.

## Conclusions

In this retrospective cohort study of anonymized patient-level pharmacy claims, coupon use for drugs treating chronic conditions was associated with the market competition facing a given drug. However, we found no association between patients’ out-of-pocket costs or neighborhood-level income and such use.

## Notes

Supplement 1. **eAppendix.** Supplemental Methods **eFigure.** Sample Selection Chart Illustrating Unique Treatment Episodes Each Step After Applying Exclusion Criteria **eTable 1.** Coupon Use Frequency by Deciles of the Proportion of Black Residents and Median Household Income in the Neighborhood **eTable 2.** Sensitivity Check: Adjusted Association of Coupon Use Frequency With Patient Characteristics by Drug Class, 2018-2019 **eTable 3.** Adjusted Association of Coupon Use Timing With Patient, Drug, and Drug-Class Characteristics, 2018-2019

Supplement 2.
Data Sharing Statement
