# Supplementary material for: Patterns of Manufacturer Coupon Use for Prescription Drugs in the US, 2017-2019
Source: JAMA Netw Open. 2023 May 16;6(5):e2313578. doi: 10.1001/jamanetworkopen.2023.13578 (PMC10189560; doi:10.1001/jamanetworkopen.2023.13578)
Supplement: Supplement 1. — eAppendix. Supplemental Methods eFigure. Sample Selection Chart Illustrating Unique Treatment Episodes Each Step After Applying Exclusion Criteria eTable 1. Coupon Use Frequency by Deciles of the Proportion of Black Residents and Median Household Income in the Neighborhood eTable 2. Sensitivity Check: Adjusted Association of Coupon Use Frequency With Patient Characteristics by Drug Class, 2018-2019 eTable 3. Adjusted Association of Coupon Use Timing With Patient, Drug, and Drug-Class Characteristics, 2018-2019 [file jamanetwopen-e2313578-s001.pdf]

Supplemental Online Content

Kang S-Y, Liu A, Anderson G, Alexander GC. Patterns of manufacturer coupon use for prescription drugs in the US, 2017-2019. *JAMA Netw Open*. 2023;6(5):e2313578. doi:10.1001/jamanetworkopen.2023.13578

eAppendix. Supplemental Methods

eFigure. Sample Selection Chart Illustrating Unique Treatment Episodes Each Step After Applying Exclusion Criteria

eTable 1. Coupon Use Frequency by Deciles of the Proportion of Black Residents and Median Household Income in the Neighborhood

eTable 2. Sensitivity Check: Adjusted Association of Coupon Use Frequency With Patient Characteristics by Drug Class, 2018-2019

eTable 3. Adjusted Association of Coupon Use Timing With Patient, Drug, and Drug-Class Characteristics, 2018-2019

This supplemental material has been provided by the authors to give readers additional information about their work.

© 2023 Kang S-Y et al. *JAMA Network Open.*

eAppendix. Supplemental Methods

Data

We performed a retrospective cohort study using IQVIA's Formulary Impact Analyzer (FIA) between October 2017 and September 2019.[11] We analyzed the data from September to December in 2022. The FIA database is a nationally representative patient-level, transactional claims database sourced from 95% of the retail pharmacies in the United States. We excluded patients enrolled in federally funded health plans (e.g., Medicare and Medicaid) since coupon use is prohibited in such plans. We focused on prescription drugs with at least one transaction where a product-specific manufacturer-sponsored coupon was used, using the method of payment and sources of the offsets to identify claims with manufacturer drug coupons as the secondary payer.[1] For each claim, these data contain a binary indicator variable for whether coupons were utilized. In addition to the binary indicator, there is a variable to identify the types of offsets and source the offset and patients' out of pocket costs before any buy down and after any buy down was applied. Transactions adjudicated with manufacturer-sponsored coupons had names of manufacturer- and/or product-specific copay programs as the sources of offset. This method was used for the previous research and validated through consultation with the data developer- IQVIA.

We supplemented the claims data with three data sources. First, we used IBM Micromedex Red Book to derive drug characteristics, including the availability of generic alternatives, and whether or not the drugs' primary indication was for an acute or chronic condition.[12] The Red Book database defines drugs treating chronic conditions as drugs with low probability for dosage or therapy changes, commonly used to treat

© 2023 Kang S-Y et al. *JAMA Network Open.*

chronic conditions, and usually administered continuously. Second, we used IQVIA's Uniform System of Classification (USC) to define the market of direct competitors based on the mechanism of action for each product of interest (e.g., dipeptidyl peptidase 4 inhibitors). The USC is IQVIA's therapeutic classification of pharmaceutical products considered to compete in the same or similar market and is widely used as the standard in North America.[2,13,14] These two data files are merged with the FIA claims data using the national drug code (NDC) for each claim line. Last, we supplemented the data with information on country income and racial characteristics obtained from the 2019 Health Resources & Services Administration Area Health Resource Files (AHRF). To merge the county-based AHRF data with the zip-code based FIA data, we assigned each individual a county of residence based on the pharmacy zip codes where most of their prescription transactions occurred.

Study sample

As per previous research, manufacturer-sponsored coupons are concentrated in a small number of drugs as reported in the prior research.[1] Consequently, many drugs did not have enough treatment episodes to observe the significant variation in coupon use patterns among patients within the drug. To address the issue, we identified the drugs that had at least 100 coupon fills reported in the data. These drugs accounted for a cumulative 80% of coupon fills. We then focused on drugs treating chronic conditions since drugs treating acute illnesses were typically administered only once, and therefore had insufficient prescription refills to observe patterns of coupon usage over the

© 2023 Kang S-Y et al. *JAMA Network Open.*

treatment episode. 113 unique drugs treating chronic conditions met this inclusion criterion.

Our primary unit of analysis was a treatment episode, which we defined as an episode of care where a product used to treat a chronic condition was initiated between January 2018 and October 2018 and that specific drug was not used during the prior 90 days. To characterize a treatment episode, we applied a 3 month "wash-out period" to capture only incident drug fills and extended the treatment episode from the time of the index date, defined as the date of the first fill, through the date at which point medication was exhausted. We required at least two fills for a given product, censored individuals with a gap of 60 days or longer without medications on hand and allowed for indefinite stockpiling.[6] The treatment episode ended if no more refills were reported with the drug for the patient or our observation period (12 months) ended.

This strategy returned 90,431 episodes with one or more manufacturer-sponsored coupons. Shorter use of drugs, such as those with only 1 or 2 fills with a coupon, accounted for 59.1% of the initial cohort. These treatment episodes may represent a drug trial or immediate drug discontinuation or drug switch. Also, including the "one"- or "two-and-done" episodes in the sample would inflate the outcome variables measuring frequency of coupon use within the treatment episode as the percentage of fills with a coupon. These analyses were focused on continuous 36,951 (40.9%) treatment episodes, which we define as treatment episodes filling 3 or more fills (eFigure).

© 2023 Kang S-Y et al. *JAMA Network Open.*

Explanatory variables

We quantified the frequency of coupon use as a function of patient, product and drug class characteristics.

We assume that patients' coupon access and utilization may be determined based on patients' need and characteristics. We considered patient characteristics, including sex, unique products filled, and the dollar level of out-of-pocket cost per claim before coupon application. We also examine whether coupons were made more accessible to lower-income patients by examining the patients' neighborhood characteristics (i.e., the median household income and percentage of black residents) in the county where the prescription drug was dispensed.[15]

Because drug companies determine the availability of coupons and may offer coupons in order to obtain a competitive advantage, we included product characteristics- single-source brand-name product and orphan drug status. In order to examine the drug's out-of-pocket cost relative to its competitors, we compared the drug out-of-pocket cost to the mean of the drugs in that therapeutic class.

Manufacturers selling single-source brand-name drugs without generic alternatives may have less incentive to offer coupons to patients. The incentive to offer coupons for orphan drugs may be different from mass market drugs, given the limited market. Conversely, manufacturers selling brand-name drugs competing with other brand-name drugs in the same therapeutic class may be sensitive to competitors' marketing and have more incentive to provide coupons. Therefore, we examined if drug companies offer coupons based on market opportunities and competition.

© 2023 Kang S-Y et al. *JAMA Network Open.*

We included the following drug class characteristics: within-class competition; mean patient cost-sharing before offset to examine if market characteristics influenced use of coupons. These variable are constructed based on the full dataset before the sample selection as reported in the prior study.[2]

To examine the level of competition, single-source and multi-source products were identified. Herfindahl-Hirschman Index (HHI),[15] an established measure of market concentration, was used to group drugs at the USC 4 level into three mutually exclusive groups: monopolistic markets (HHI ≥ 8000),oligopolistic markets (≥ 2500 and < 8000), and competitive markets (< 2500). Additional variables examining the market characteristics were incorporated into the models including manufacturer characteristics (i.e., revenue size) and total market variables for that therapeutic category.

Outcome variables

We examined two outcomes. The first outcome of interest was the frequency of coupon use during a given treatment episode, which we assessed as the proportion of prescription fills purchased with a coupon out of the total number of fills within the treatment episode. In descriptive analyses, we dichotomized treatment episodes into two groups, infrequent coupon use (coupons used for less than two-thirds of treatment episode fills) and frequent coupon use (more than two-thirds of fills). In the multivariate regression examining characteristics associated with coupon use, this outcome was treated as a continuous measure.

The second outcome of interest was the timing of the first coupon use within the treatment episode to examine if coupons are used to fulfill the prescription for the new treatment, which is known as a measure of primary drug adherence.[16] In examining this

© 2023 Kang S-Y et al. *JAMA Network Open.*

outcome, we dichotomized individuals into those using a coupon during the first fill of their treatment episode and those who did not.

eFigure. Sample Selection Chart Illustrating Unique Treatment Episodes Each Step After Applying Exclusion Criteria

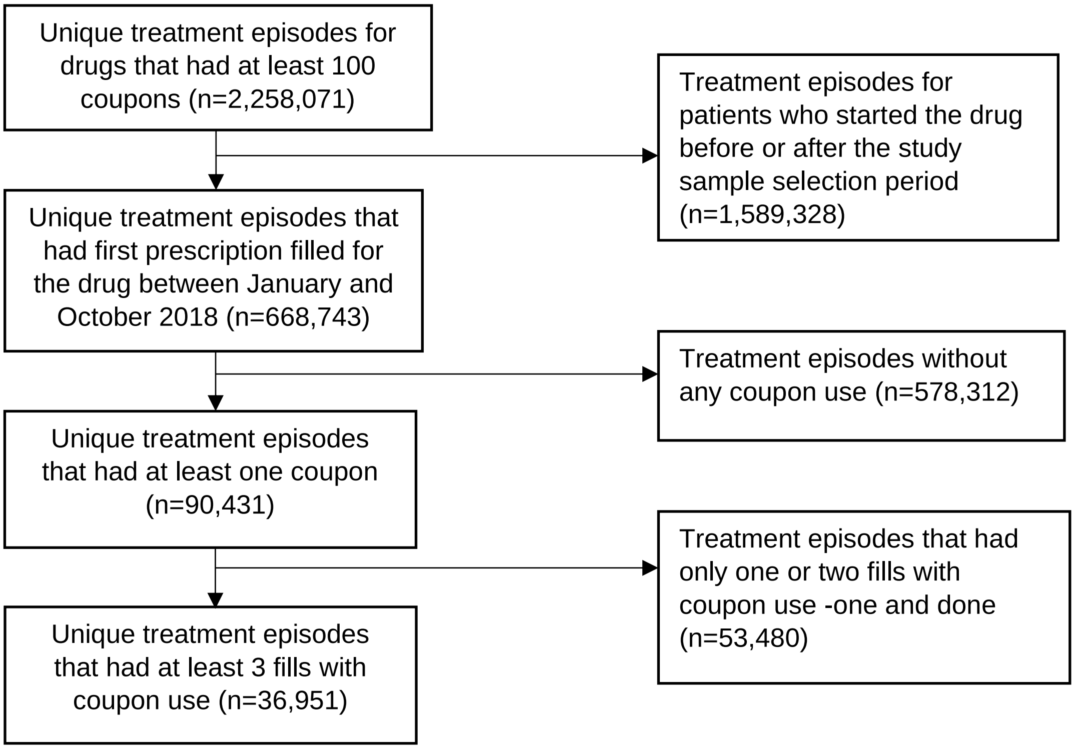

© 2023 Kang S-Y et al. *JAMA Network Open.*

eTable 1. Coupon Use Frequency by Deciles of the Proportion of Black Residents and Median Household Income in the Neighborhood

	All coupon users	Frequent coupon users,<br>Share of treatment episodes, %
Deciles by proportion of Black residents		
1st decile	61.0 (32.5)	82.0 (21.5), 59.3%
2nd	63.9 (32.0)	83.5 (20.6), 62.0%
3rd	65.2 (31.8)	83.8 (20.9), 63.6%
4th	66.0 (31.6)	84.0 (20.6), 64.8%
5th	65.3 (31.8)	84.5 (20.2), 62.5%
6th	66.5 (31.5)	84.6 (20.2), 64.9%
7th	66.1 (31.2)	84.0 (20.6), 64.9%
8th	66.9 (31.3)	84.9 (20.3), 64.5%
9th	68.4 (30.8)	85.0 (20.2), 66.6%
10th decile	68.1 (31.0)	84.6 (20.3), 67.1%
Deciles by income		
1st decile	62.7 (32.4)	82.8 (21.0), 61.6%
2nd	63.7 (32.2)	83.6 (20.6), 62.0%
3rd	65.5 (31.7)	83.5 (20.9), 64.7%
4th	65.1 (31.9)	83.7 (20.9), 63.4%
5th	64.4 (31.5)	82.9 (21.1), 62.5%
6th	66.8 (31.4)	84.6 (20.3), 65.1%
7th	67.0 (31.3)	84.6 (20.2), 65.3%
8th	67.2 (31.4)	84.5 (20.6), 66.0%
9th	67.1 (30.9)	84.6 (20.5), 64.3%
10th decile	68.1 (30.9)	85.7 (19.5), 65.4%

Source: Authors' analysis of IQVIA Formulary Impact Analyzer, 2018-2019

© 2023 Kang S-Y et al. *JAMA Network Open.*

eTable 2. Sensitivity Check: Adjusted Association of Coupon Use Frequency With Patient Characteristics by Drug Class, 2018-2019

	(1)	(2)	(3)	(4)	(5)	(6)
	M1: Primary regression -- all drugs	M2: Regression subset to acne treatments without anti-infectives	M3: Regression subset to HIV antiviral combinations	M4: Regression subset to ophthalmic immunologic	M5: Regression subset to inhaled bronchial corticosteroids	M6: Regression subset to sodium-glucose co-transporter 2 inhibitors
	Proportion of fills with coupon use	Proportion of fills with coupon use	Proportion of fills with coupon use	Proportion of fills with coupon use	Proportion of fills with coupon use	Proportion of fills with coupon use
Patients using 2 or more drugs	-4.162***	-5.547	-12.99***	-9.646*	-4.723*	-5.659***
	(0.705)	(4.003)	(2.582)	(3.848)	(2.356)	(1.399)
Patient age	-0.00296**	0.00419	-0.000201	-0.00660	-0.0104**	-0.00452
	(0.00101)	(0.00485)	(0.00837)	(0.00430)	(0.00340)	(0.00428)
Patient sex (reference: male)	-0.230	-1.197	-9.057***	2.045	0.224	-0.180
	(0.419)	(0.948)	(1.916)	(1.890)	(1.053)	(0.944)
Neighborhood-level income	-0.0710	0.188	0.358	-0.520	0.209	0.0986
	(0.0685)	(0.178)	(0.209)	(0.271)	(0.188)	(0.167)
Share of Black residents	-0.140*	0.296	0.444	-0.432	-0.0971	-0.255
	(0.0699)	(0.180)	(0.246)	(0.277)	(0.188)	(0.169)

© 2023 Kang S-Y et al. *JAMA Network Open.*

eTable 3. Adjusted Association of Coupon Use Timing With Patient, Drug, and Drug-Class Characteristics, 2018-2019

	Incident fill with coupon	
	Odds Ratio (95% Confidence Interval)	*p*-value
Patient Characteristics		
Patients using 3 or more drugs	0.9 (0.8-1.0)	0.03
Patient copay per claim before coupon	1.1 (1.1-1.1)	<0.001
Drug Characteristics		
Single-source brand-name drugs	1.7 (1.4-2.1)	<0.001
Orphan designation status	1.2 (1.0-1.5)	0.05
Drug-class Characteristics		
In-class mean copay per claim	1.9 (1.2-2.9)	<0.001
Oligopolistic market	1.6 (0.9-2.8)	0.091
Competitive market	3.4 (1.4-8.1)	0.01

Source: IQVIA Formulary Impact Analyzer, 2018-2019

Note. Estimates are derived from a multilevel regression model of the coupon use frequency among commercially insured patients to account for clustering by drug class. See Methods for further details.

© 2023 Kang S-Y et al. *JAMA Network Open.*
