# Supplementary material for: Patterns of Manufacturer Coupon Use for Prescription Drugs in the US, 2017-2019
Source: JAMA Netw Open. 2023 May 16;6(5):e2313578. doi: 10.1001/jamanetworkopen.2023.13578 (PMC10189560; doi:10.1001/jamanetworkopen.2023.13578)
Supplement: Supplement 2. — Data Sharing Statement [file jamanetwopen-e2313578-s002.pdf]

Data Sharing Statement

Kang. Patterns of Manufacturer Coupon Use for Prescription Drugs in the US, 2017-2019. *JAMA Netw Open*. Published May 16, 2023. doi:10.1001/jamanetworkopen.2023.13578

Data
Data available: No
